# Diode laser *versus* scalpel in the surgical treatment of infant ankyloglossia: a randomized, parallel, double-blind, controlled clinical trial

**DOI:** 10.1186/s12887-025-06307-y

**Published:** 2025-12-04

**Authors:** Mariana Laprovitera Teixeira Carneiro, Maria Elisa Quezado Lima Verde, Maria Fernanda da Silva Nascimento, João Emanuel Sousa de Almeida, Maria Clara Mendes Gomes, Rebeca Neneza Dias Barboza, Sheyla Cristinty Rodrigues Mendes, Laís Fernandes Pontes Mendonça, Luzia Rayane Gomes de Lima, Barbhara Girão Costa Rodrigues, Gustavo Mendes de Oliveira, Paulo Goberlânio de Barros Silva, Juliana Ximenes Damasceno

**Affiliations:** 1 Postgraduate Program in Dental Sciences, Christus University Center (Unichristus), Fortaleza, Ceará Brazil; 2 Department of Surgery and Oral Medicine, Faculty of Dentistry, Christus University Center (Unichristus), Fortaleza, Ceará Brazil; 3 Faculty of Dentistry, Christus University Center (Unichristus), Fortaleza, Ceará Brazil; 4 Department of Pediatric Dentistry, Faculty of Dentistry, Christus University Center (Unichristus), Fortaleza, Ceará Brazil

**Keywords:** Laser therapy, Ankyloglossia, Infant

## Abstract

**Background:**

Ankyloglossia can impair vital functions such as breastfeeding, swallowing, and maxillomandibular development, making effective treatment essential. This study aimed to compare the clinical effectiveness of diode laser (DL) and scalpel (SC) frenotomy in infants with severe ankyloglossia.

**Methods:**

This randomized, parallel, double-blind clinical trial evaluated the efficacy of diode laser surgery compared to conventional scalpel frenotomy in infants aged 0–6 months, all diagnosed with severe ankyloglossia (BTAT score 0–3) (*n* = 40/group). Patients were assessed at D0 (pre-, trans-, and/or 30 min postoperative), D7, and D14 using specific instruments according to each outcome: tongue repositioning and frenulum release (BTAT and Tongue Test), wound healing (Landry index), perioperative pain (CRIES scale), and breastfeeding performance (body mass variation, LATCH, and IBFAT scales).

**Results:**

Both the diode laser (DL) and scalpel (SC) groups showed significant functional improvements and weight gain over time (*p* < 0.001), with no significant intergroup differences in most outcomes (*p* > 0.05). However, on D7, the SC group demonstrated superior breastfeeding performance (LATCH: *p* = 0.003; IBFAT: *p* = 0.024). Both groups exhibited reduced pain and progressive healing (*p* < 0.001). In the diode laser group, a negative correlation was found between total energy applied and healing quality at D14 (*r* = − 0.355; *p* = 0.027).

**Conclusions:**

Findings suggest both techniques are clinically effective, but laser parameters may influence healing. Further studies are warranted to optimize laser protocols in pediatric ankyloglossia surgery.

**Trial registration:**

RBR466cykz November 13th, 2024.

## Background

The lingual frenulum is a fibrous connective tissue structure that connects the ventral surface of the tongue to the floor of the mouth [1]. Along with the labial frenulum, it plays a key role in the function of the lingual and perioral muscles, being essential for speech, swallowing, sucking, and breathing. Ankyloglossia, or “tongue-tie,” is a congenital condition characterized by a short or thickened lingual frenulum that restricts tongue mobility [2]. This alteration results from insufficient apoptosis during embryonic development and may occur sporadically or be associated with craniofacial anomalies. Genetic inheritance patterns, including autosomal dominant and recessive forms, have also been described. Ankyloglossia is more prevalent in males, with a reported male-to-female ratio of approximately 2.5:1 [3].

Clinically, ankyloglossia can lead to breastfeeding difficulties, speech and feeding disorders, malocclusion, impaired oral hygiene, and psychosocial stress. Prevalence estimates vary widely (0.52%–21%) due to heterogeneity in diagnostic criteria and methodologies [4]. Several diagnostic tools have been proposed, such as the Coryllos classification (which focuses on anatomical presentation) and functional assessment protocols including the Hazelbaker Assessment Tool for Lingual Frenulum Function (HATLFF), the Bristol Tongue Assessment Tool (BTAT), and the Lingual Frenulum Assessment Protocol for Infants (“Tongue Test”). Although the Brazilian Ministry of Health recommends the BTAT for infants, there is no global consensus on a gold-standard protocol. Recent studies have presented conflicting findings, with some reporting greater sensitivity for the “Tongue Test,” while others support the BTAT as a simple, objective, and effective tool for both diagnosis and post-intervention follow-up [5].

Despite increased awareness and diagnosis of ankyloglossia in recent years, the benefits of surgical intervention—particularly in infants—remain under debate. Professional societies, such as the Academy of Breastfeeding Medicine and the American Society of Pediatric Otolaryngology, have highlighted the lack of robust randomized clinical trials and standardization in diagnostic and treatment criteria. As a result, overdiagnosis and potentially unnecessary surgical procedures have been reported in certain populations [6, 7].

Breastfeeding complications in the neonatal period are common, and ankyloglossia may contribute to problems such as poor latch, maternal nipple pain, breast engorgement, and reduced milk supply. Nevertheless, these symptoms may also result from other factors. Thus, multidisciplinary evaluation is crucial in distinguishing ankyloglossia from other breastfeeding issues and in supporting clinical decision-making [8].

When conservative management fails to resolve breastfeeding difficulties, frenotomy—defined as the simple incision of the lingual frenulum—is the most indicated procedure in infants due to its simplicity, low morbidity, and immediate potential benefits. In older children, frenuloplasty, which involves muscle release and suturing, may be preferred, while frenectomy is reserved for cases requiring tissue excision [8, 9]. Surgical options include conventional blade or scissors, electrocautery, and different lasers [10, 11].

High-power lasers (e.g., diode, Nd: YAG) offer several advantages: enhanced hemostasis, microvascular sealing, precision cutting, better visualization of the operative field, reduced postoperative pain and inflammation, and faster tissue repair. These effects are attributed to photothermal interactions, including vaporization, coagulation, and necrosis [12]. However, cost and the need for training may limit widespread use. Among laser technologies, diode lasers have shown a particular promise in pediatric applications due to their safety and efficacy [13, 14].

Studies comparing laser and conventional techniques report benefits such as reduced anesthesia need, improved cooperation in children, lower postoperative pain, and better maternal comfort during breastfeeding [15]. Laser use has also been associated with decreased bleeding and inflammation compared to electrocautery [14].

Despite these promising results, the literature lacks well-designed randomized clinical trials evaluating the efficacy and safety of diode lasers in treating ankyloglossia in infants. Limitations include small sample sizes, heterogeneous diagnostic criteria, variable surgical protocols, and reliance on qualitative outcomes. Therefore, this study aimed to evaluate the clinical effectiveness of diode laser surgery compared to the conventional scalpel technique in the treatment of pediatric ankyloglossia.

## Methods

### Study design and participants

The research protocol was approved by the Human Research Ethics Committee of Christus University Center under approval number 6.332.477. This study was conducted in accordance with the Declaration of Helsinki, the Brazilian National Health Council’s Resolution 466/2012 and the federal law 14.874/2024, which outlines ethical principles such as autonomy, non-maleficence, beneficence, justice, and equity. Guardians of the participants were informed about the objectives, procedures, potential risks, and benefits. Written informed consent was obtained from each legal guardian prior to their participation. They were also informed of their right to withdraw at any time without any consequences.

This randomized, controlled, parallel, double-blind clinical trial was registered on the Brazilian Clinical Trials Registry (ReBEC) under registration number RBR-466cykz and conducted in accordance with the CONSORT 2010 guidelines.

Infants were recruited from spontaneous demand at the School Clinic of Dentistry, Christus University Center, where eligibility screening and all surgical procedures were performed. All patients were referred by speech-language pathologists, nurses, or lactation consultants due to reported functional breastfeeding limitations.

Following referral, infants underwent a morphological evaluation using the Bristol Tongue Assessment Tool (BTAT), a validated, rapid, and reproducible instrument recommended by the Brazilian Ministry of Health in Nota Técnica nº 35/2018 as the preferred morphological assessment tool for early detection of ankyloglossia in newborns, complementary to functional assessment.

Only those with BTAT scores between 0 and 3, indicating more severe anatomical restriction, were included in the study.

**Fig. 1:**
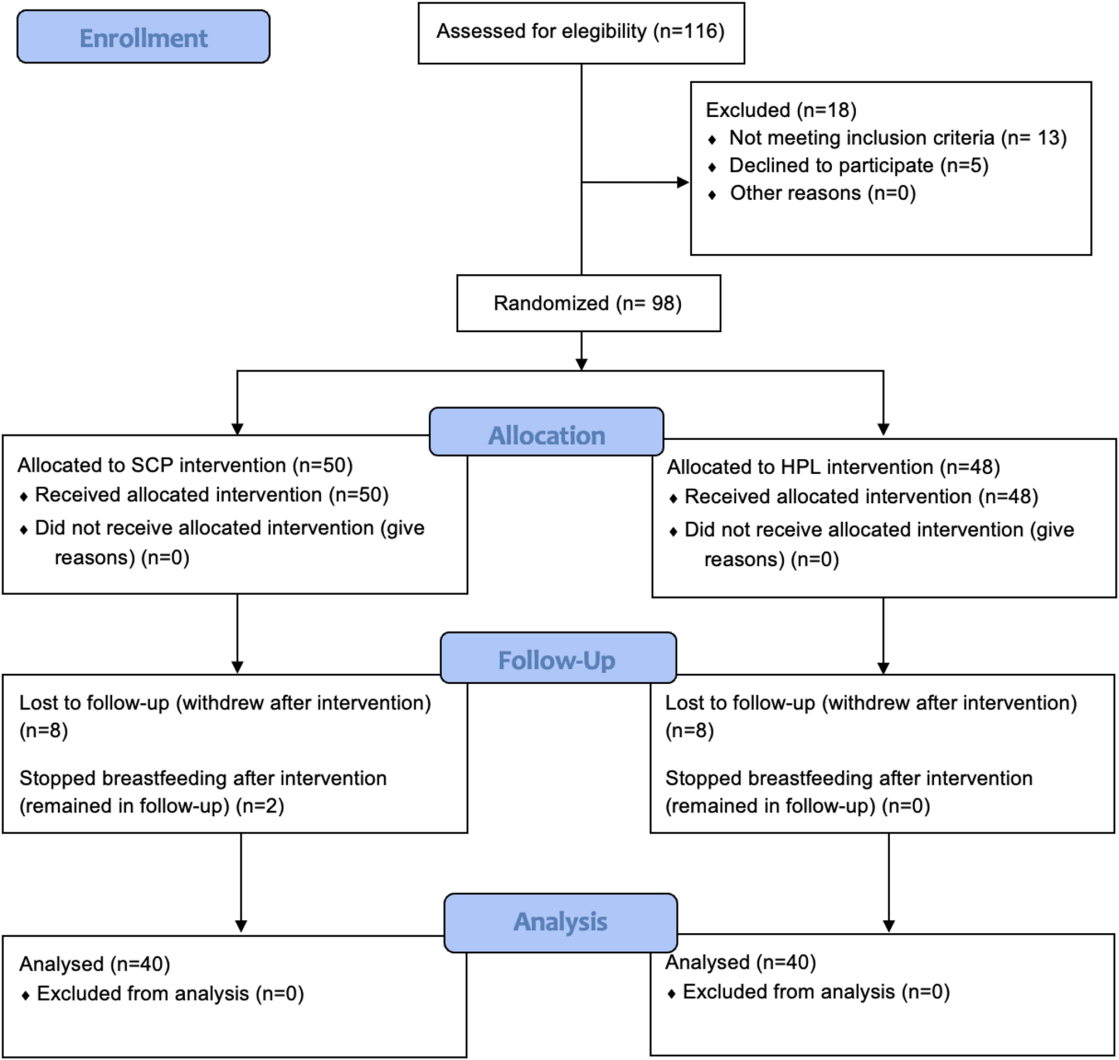
CONSORT flow diagram of participant enrollment, allocation, follow-up, and analysis. Final analysis included 40 participants per group

Participants included in this study were healthy individuals (ASA I), aged between 0 and 6 months, diagnosed with severe ankyloglossia by a pediatric dentist using the BTAT protocol, with scores ranging from 0 to 3. Exclusion criteria comprised individuals with previously diagnosed systemic conditions that contraindicated the surgical procedure; those undergoing prior pharmacological treatment; individuals with absolute or relative contraindications to the use of local anesthetics; those with congenital orofacial malformations; and cases in which the legal guardians refused to consent to participation in the study. Participants were withdrawn from the study if they failed to attend postoperative follow-up appointments, if postoperative care instructions were not followed, or if their guardians chose to discontinue participation (Fig. 1).

### Sample size calculation

Based on the study by BHAT et al. in 2015, which compared lingual frenulum removal via scalpel (1.12 ± 0.19 mm³) and high-power laser (−1.3 ± 0.12 mm³) and reported a significant reduction in remnant tissue volume comparing scalpel and high-power laser, t test was used to perform a sample size calculation. So, a sample size of 40 patients per group was calculated to ensure 80% power and 95% confidence (Student’s t-test) [16].

Participants were randomly allocated into two groups: SCP group: incision performed with a no. 15 scalpel blade; and HPL group: incision performed with high-power diode laser.

### Randomization and blinding

Simple randomization was performed using Microsoft Excel^®^ (version 16.73) via the “=RANDBETWEEN” function. A study assistant uninvolved in any research procedures generated the random sequence and allocated participants into groups. Group assignment was revealed to the surgeon only at the time of the procedure.

To ensure double blinding, participant/guardian blinding and blinded outcome assessment and analysis were performed. Guardians remained immediately outside the procedure room and could enter at any time if they wished or if they felt anxious, ensuring transparency and emotional support. This arrangement was explained during the consent process, and no guardian requested to remain in the room during the procedure. As the surgical techniques inevitably differed between groups, the surgeon could not be blinded. To minimize operator-related bias, measures included a standardized operative protocol, predefined intraoperative criteria, and a clear separation between surgical care and outcome assessment/analysis. Statistical analyses were initially performed using coded group labels (‘A’ and ‘B’), and group identities were revealed only after completion of all analyses.

### Interventions

All surgical procedures were performed by a single experienced surgeon, while all pre- and postoperative assessments were carried out by a single experienced pediatric dentist, ensuring standardization and blinding. The diagnosis of severe ankyloglossia was established during the clinical examination using the BTAT protocol (scores from 0 to 3). Topical anesthesia with 20% benzocaine was followed by infiltrative anesthesia using 2% lidocaine with epinephrine (1:100,000), adjusted according to the infant’s weight [3].

In Brazil, benzocaine is not expressly prohibited for use in children, but it should be used with caution and only under medical prescription in certain age groups. In this study, a minimal dose was applied with gauze under continuous aspiration with the suction tip. All patients were closely monitored for potential allergic reactions, changes in blood coloration, cyanosis or any other signs suggestive of methemoglobinemia, airway obstruction or excessive bleeding, with readiness to manage possible adverse events (administration of oxygen, airway clearance maneuvers, use of suction equipment, hemostasis with sterile gauze compression, and readiness for pharmacological intervention or advanced airway management if necessary). Our decision to include benzocaine in the protocol was adapted from another brazilian study [3], which used two topical anesthetics followed by infiltrative anesthesia with 3% mepivacaine. It is important to consult the regulations of each country and carefully weigh the risks and benefits of benzocaine use in pediatric population, considering national guidelines, patient age, and the known risk of methemoglobinemia.

With the aid of a tongue retractor (tentacannula), the patient’s tongue was pulled posteriorly to achieve stable tension of the lingual frenulum, allowing for a favorable incision site. In the SCL group, a no. 15 scalpel blade mounted on a no. 3 Bard Parker handle was used. In the DL group, a high-power diode laser (Thera Lase Surgery^®^, DMC, São Carlos, Brazil) operating at 980 ± 20 nm (infrared region), in continuous mode with a power of 1.5 W, was used for all patients. The laser was coupled to a 400 μm optical fiber. Before the procedure, the fiber tip was cleaved using a diamond-tipped pen [14, 17].

In both groups, the incision extended from the anterior border of the frenulum to its posterior-inferior limit, avoiding noble anatomical structures. Additional hemostasis, when necessary, was first achieved with sterile gauze compression; suturing of the raw surfaces was considered only if bleeding could not be controlled by gauze pressure alone. In line with existing literature, bleeding in infant frenotomy is typically scant and readily controlled by compression, and the incision usually does not require suturing [9]. Guardians received written and verbal postoperative instructions, including administration of oral paracetamol (100 mg/ml in oral suspension), when necessary, wound care, feeding recommendations, and signs of possible complications to monitor. Patients were followed up during the 14-day healing period and, after complete healing, were referred to a speech-language pathologist for guidance and supervision of functional tongue exercises to prevent recurrence and optimize mobility.

#### Harms and benefits of the intervention

The procedure carried some risks, such as pain, edema, bleeding, paresthesia, ulcers, and infection. However, in case of complications, patients were reassessed and managed accordingly. On the other hand, benefits of ankyloglossia treatment include improvements in breastfeeding and further in speech, nutrition, and quality of life.

### Outcome evaluation

The primary outcome of the study was the release and repositioning of the lingual frenulum. Secondary outcomes included: perioperative pain; healing quality; variation in the patient’s body weight and quality of breastfeeding. The evaluation of these outcomes was based on the following methodologies:

#### Release and repositioning of the lingual frenulum

Assessment was conducted using the BTAT and Tongue Test scales at baseline preoperative (D0-pre) and immediate postoperative (D0-post), day 7 (D7), and day 14 (D14) [3, 18]. Data were presented as mean ± standard deviation (SD) for both groups. Standardized photographs were taken at each time point.

#### Impact on the healing process

Postoperative healing was evaluated quantitatively at D0-post, D7, and D14 using the Landry Healing Index [18], which scores five criteria—bleeding, granulation tissue, tissue color, wound margins, and suppuration—on a scale from 0 (poor healing) to 5 (excellent healing). Results were expressed as mean ± SD.

#### Perioperative pain

Pain was assessed using the CRIES scale, which scores five parameters (crying, oxygen requirement, vital signs, facial expression, and sleepiness) from 0 to 10 [18]. Evaluations were conducted by the same examiner at three time points on D0 (intraoperative (D0-intra); immediate postoperative (D0-post) and 30 min post-operative (D0-30post)), as well as on D7 and D14. A pulse oximeter was used as part of the assessment and results were expressed as mean ± SD. Rescue medication usage (paracetamol) was recorded and reported as absolute and relative frequency.

#### Variations in the patient’s body mass

Patients’ body weight was measured using a digital scale on D0, D7, and D14. Data were presented as mean ± SD for each group.

#### Quality of breastfeeding

Breastfeeding performance was assessed using two validated tools: the LATCH (Latch, Audible swallowing, Type of nipple, Comfort, Hold) breastfeeding assessment tool and the IBFAT (Infant Breastfeeding Assessment Tool). Both instruments are widely used in clinical and research contexts to evaluate latch quality, infant behavior, and feeding effectiveness [19]. Assessments were performed by a blinded pediatric dentist through direct observation of breastfeeding and standardized maternal feedback during the evaluation, ensuring a reproducible and structured measurement of feeding outcomes.

LATCH scale contains five scored items (0–2 each), totaling up to 10 points; and IBFAT four scored items (0–3 each), totaling up to 12 points [20]. Assessments were performed at D0-pre, D0-post, D7, and D14. Results were expressed as mean ± SD.

### Statistical analysis

All data were exported to IBM^®^ SPSS^®^ Statistics for Windows^®^ (version 20) for analysis. A confidence level of 95% (*p* < 0.05) was adopted for all tests. The normality of the data was assessed using the Kolmogorov-Smirnov test. Continuous variables were expressed as mean ± SD and analyzed using the Mann-Whitney and Friedman tests, followed by Dunn’s post hoc test for non-parametric data. Categorical variables were presented as absolute and relative frequencies and compared using either Fisher’s Exact Test or Pearson’s Chi-square test, as appropriate. Additionally, the correlation between the total amount of energy applied (in Joules) in the DL group and the Landry wound healing index across different time points was evaluated using Spearman’s correlation coefficient.

## Results

### Demographic characteristics

Ninety-eight infants met the inclusion criteria and were enrolled in the study. The SC group included 50 patients (51.02%), with 10 subsequently withdrawn due to loss to follow-up (*n* = 8) or discontinuation of breastfeeding (*n* = 2). The DL group consisted of 48 patients (48.98%), with 8 excluded due to missed follow-up visits. Both groups were similar in terms of age, sex distribution, type of breastfeeding, and use of artificial nipples (Tables 1 and 2).

**Table 1:** Description of clinical data (gender and age) in the different groups evaluated

	SC	DL	*p*-Value
Sex			0,068
Female	20 (50.0%)	12 (30.0%)	
Male	20 (50.0%)	28 (70.0%)	
Age (days)	46.73 ± 31.76	42.78 ± 38.23	0,331

*SC* Group treated with scalpel, *DL* Group treated with diode laser

*p < 0.05, Mann-Whitney test (mean ± SD) and Pearson’s chi-square (n, %)

**Table 2:** Description of the types of breastfeeding and use of artificial nipples, according to the evaluation groups

	SC	DL	*p*-Value
Breastfeeding			
Exclusive	28 (70.0%)	25 (62.5%)	0,478
Associated with the formula	12 (30.0%)	14 (35.0%)	0,633
Associated with food introduction	1 (2.5%)	1 (2.5%)	1,000
Use of artificial nipples	26 (65.0%)	32 (80.0%)	0,133
Silicone nozzle	17 (42.5%)	13 (32.5%)	0,356
Baby bottle	17 (42.5%)	21 (52.5%)	0,370
Pacifiers	7 (17.5%)	12 (30.0%)	0,189

*SC* Group treated with scalpel, *DL* Group treated with diode laser

*p < 0.05, Pearson’s chi-square test (n, %)

The clinical profile, including breastfeeding types and use of artificial nipples, did not differ significantly between groups. Regarding clinical characteristics, no statistically significant difference was observed between groups in terms of gender (*p* = 0.068), although the DL group included a higher proportion of males (70%) compared to the SC group (50%). The mean age of participants was also similar across groups (SC: 46.73 ± 31.76 days; DL: 42.78 ± 38.23 days; *p* = 0.331) (Table 1).

Given that the study included infants who could be exclusively breastfed or supplemented with formula and/or solid food, most participants in both groups were exclusively breastfed (SC: 70%, DL: 62.5%), with no significant difference (*p* = 0.478). No significant differences were found in the combination of breastfeeding with formula (*p* = 0.633) or with the introduction of solid foods (*p* = 1.000) (Table 2).

The use of artificial nipples was more frequent in the DL group (80%) compared to the SC group (65%), but this difference was not statistically significant (*p* = 0.133). Similarly, no significant differences were observed between groups in the use of specific types of artificial nipples such as silicone nipples (*p* = 0.356) or baby bottles (*p* = 0.370). Pacifier use was also more prevalent in the DL group (30% vs. 17.5% in the SC group), but the difference was not statistically significant (*p* = 0.189) (Table 2).

### Clinical outcomes

Morphological and functional assessments were conducted at four time points (D0-pre, D0-post, D7, and D14) using two validated tools: the BTAT and the Tongue Test. No significant differences were observed between groups on any assessment day regardless of the assessment method used (*p* > 0.05) (Table 3).

**Table 3:** Assessment of tongue release and repositioning (BTAT and tongue Test) at the different assessment times, according to the treatment modality

	SC	DL	*p*-Value
BTAT			
D0-pre	2.45 ± 0.81	2.45 ± 0.81	1,000
D0-post	4.38 ± 1.05*	4.63 ± 0.95*	0,180
D7	4.93 ± 1.05*†	5.03 ± 1.14*	0,631
D14	5.15 ± 1.21*†	5.40 ± 1.45*	0,298
p-Value	***< 0***,***001***	***< 0***,***001***	
Tongue test			
D0-pre	13.65 ± 3.71	13.93 ± 3.38	0,828
D0-post	9.77 ± 3.21*	9.79 ± 3.16*	0,956
D7	9.25 ± 3.15*	10.23 ± 3.29*	0,260
D14	7.90 ± 3.46*†	8.58 ± 3.05*†	0,312
p-Value	***< 0***,***001***	***< 0***,***001***	

*SC* Group treated with scalpel, *DL* Group treated with diode laser, *D* Day

*p < 0.05 versus D0; †*p* < 0.05 versus previous day; Friedaman/Dunn or Mann-Whitney test (mean ± SD)

Regarding the BTAT assessment, a significant improvement in tongue morphology was observed over time in both groups. In the SC group, significant differences emerged immediately after surgery (D0-post) compared to D0-pre, with further improvement on D7 and D14 (*p* < 0.001). Similarly, in the DL group, a significant increase was seen on D0-post compared to D0-pre, with consistent values maintained on D7 and D14 (*p* < 0.001). Despite this intra-group progression, no significant inter-group differences were detected at any time point (*p* > 0.05) (Table 3).

Similarly, the Tongue Test also revealed significant improvements in both morphological and functional tongue release. Both groups demonstrated significant gains on D0 post (vs. D0 pre), stable scores on D7, and further improvement on D14 (*p* < 0.001). The parameters remained similar on the 7th day of postoperative follow-up and, on the 14th day of evaluation, increased significantly in both groups (*p* < 0.001) (Table 3).

Body mass was assessed on D0-pre, D7, and D14. No significant differences were observed between groups at any time point (D0-pre: *p* = 0.064; D7: *p* = 0.100; D14: *p* = 0.131). However, within-group analysis revealed a significant increase in body mass over time. Both the SC and DL groups showed a statistically significant increase from D0 to D7, and again from D7 to D14 (*p* < 0.001) (Table 4).

**Table 4:** Description of the data on body mass variations on the day of surgery and on the days of post-operative follow-up, according to the treatment modality performed

Body mass (Kg)	SC	DL	*p*-Value
D0	5.03 ± 1.27	4.54 ± 1.19	0,064
D7	5.30 ± 1.23*	4.90 ± 1.25*	0,100
D14	5.60 ± 1.25*†	5.18 ± 1.27*†	0,131
p-Value	***< 0***,***001***	***< 0***,***001***	

*SC* Scalpel-treated group, *DL* Diode laser-treated group, *D* Day

*p < 0.05 versus D0; †*p* < 0.05 versus previous day; Friedman/Dunn or Mann-Whitney test (mean ± SD)

Breastfeeding quality was assessed using the LATCH and IBFAT scales, in which higher scores indicate better outcomes.

No significant differences were observed between groups at D0 (pre- and post-surgery) or D14 (*p* > 0.05). However, on D7, the SC group showed significantly better performance compared to the DL group in both LATCH (*p* = 0.003) and IBFAT (*p* = 0.024) assessments (Table 5).

**Table 5:** Assessment of breastfeeding quality using the LATCH and IBFAT protocols at the different assessment times, according to the treatment modality used

	SC	DL	*p*-Value
LATCH			
D0-pre	8.40 ± 1.52	8.15 ± 1.82	0,640
D0-post	8.88 ± 1.64*	8.75 ± 2.08*	0,898
D7	9.70 ± 0.52*	8.83 ± 1.82*	***0***,***003***
D14	9.50 ± 0.88*	9.03 ± 1.56*	0,162
p-Value	***< 0***,***001***	***0***,***007***	
IBFAT			
D0-pre	10.53 ± 1.45	11.00 ± 1.15	0,131
D0-post	10.10 ± 2.84	10.45 ± 2.93	0,356
D7	11.50 ± 0.78*	10.65 ± 2.17	***0***,***024***
D14	11.08 ± 1.99*	10.48 ± 2.81	0,486
p-Value	***0***,***001***	0,888	

*SC* Group treated with scalpel, *DL* Group treated with diode laser, *D* Day, *LATCH* Latch, audible swallowing, type of nipple, comfort, hold, *IBFAT* Infant Breastfeeding Assessment Tool

*p < 0.05 versus D0; †*p* < 0.05 versus previous day; Friedaman/Dunn or Mann-Whitney test (mean ± SD)

In the SC group, LATCH scores improved significantly from D0-post and remained stable thereafter (*p* < 0.001). In the DL group, the improvement was also significant, though less pronounced (*p* = 0.007).

Regarding IBFAT, significant improvement was seen only in the SC group beginning on D7 (*p* = 0.001). The DL group did not show statistically significant changes in IBFAT scores over the assessment period (Table 5).

Pain was assessed using the CRIES scale and the need for rescue analgesia. No significant differences were found between groups on any evaluation day (*p* > 0.05), indicating a similar pain experience (Table 6). Likewise, healing outcomes did not differ significantly between the SC and DL groups (*p* > 0.05).

**Table 6:** Analysis of perioperative pain parameters (CRIES), need for postoperative analgesic medication and landry’s healing index at the different evaluation times, according to the treatment modality performed

	SC	DL	*p*-Value
CRIES scale			
D0-transoperative	4.75 ± 1.60	4.77 ± 1.46	0,674
D0-immediate post	3.80 ± 1.95*	4.03 ± 1.79*	0,637
D0-after 30 min	1.88 ± 1.65*†	1.58 ± 1.38*†	0,442
D7	2.58 ± 1.69*†	2.43 ± 1.66*†	0,910
D14	2.50 ± 1.47*†	2.78 ± 1.83*†	0,523
p-Value	***< 0***,***001***	***< 0***,***001***	
Use of post-operative analgesia	20 (50.0%)	25 (62.5%)	0,260
Landry’s healing index			
D0	3.78 ± 0.42	3.73 ± 0.51	0,750
D7	3.92 ± 0.58	3.98 ± 0.62	0,707
D14	4.80 ± 0.52*†	4.82 ± 0.45*†	1,000
p-Value	***< 0***,***001***	***< 0***,***001***	

*SC* Group treated with scalpel, *DL* Group treated with diode laser, *D* Day, *LATCH* Latch, audible swallowing, type of nipple, comfort, hold, *IBFAT* Infant Breastfeeding Assessment Tool

*p < 0.05 versus D0; †*p* < 0.05 versus previous day; Friedaman/Dunn or Mann-Whitney test (mean ± SD)

In both groups, the CRIES scores showed a significant reduction from the intraoperative to the immediate postoperative period (*p* < 0.001), with further reduction at 30 min post-surgery. Pain levels remained low and stable on subsequent days (Table 6).

### Surgical outcomes

**Fig. 2:**
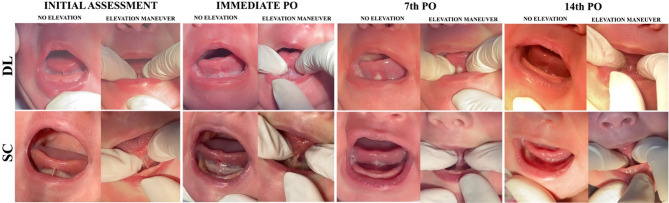
Clinical progression of lingual frenulum appearance following frenotomy with diode laser (DL) and scalpel (SC) at initial assessment, immediate postoperative (PO), 7th postoperative day, and 14th postoperative day, shown both without and with the tongue elevation maneuver

Bleeding was minimal in all cases and controlled intraoperatively with sterile gauze compression, when necessary. No patient experienced postoperative bleeding, infection or reattachment, and cicatrization occurred by secondary intention in all patients, as the wounds were intentionally left unsutured (Fig. 2).

Healing was assessed using the Landry Healing index. A significant improvement in wound healing was observed in both groups on D14 compared to D0 and D7 (*p* < 0.001) (Table 6).

In the DL group, a statistically significant negative correlation (*r* = − 0.355; *p* = 0.027) was observed between the total energy applied (in Joules) and the Landry Healing Index on D14 (Table 7), meaning that a higher intraoperative energy is associated with less favorable healing after 14 days.

**Table 7:** Correlation analysis between the total amount of energy (J) and landry’s healing index at the different assessment times

DL		Landry’s healing index		
Energy (J)		D0	D7	D14
	r	0.189	−0.025	−0.355*
	p-Value	0.243	0.877	**0.027**

*DL* Group treated with diode laser, *D* Day

*p < 0.05, Spearman’s correlation

## Discussion

This study compared two surgical techniques, scalpel (SC) and diode laser (DL), for the treatment of ankyloglossia in infants. The patient sample was clinically homogeneous, with similar distributions in gender, age, breastfeeding type, and artificial nipple use, minimizing potential confounding variables.

The assessment of tongue release and repositioning using two protocols (BTAT and Tongue Test) aimed to reduce any diagnostic and postoperative assessment bias. The BTAT, used in initial enrollment, provided a simplified and objective approach and has been validated as a reliable predictor of breastfeeding difficulties in infants aged 0–6 months [21].

Results demonstrated that both SC and DL were effective in improving tongue morphology and function across all time points, with no statistically significant differences between groups. The BTAT scores showed significant improvement in both groups, though their progression patterns differed: SC exhibited a continuous increase in scores, while DL showed a plateau after initial improvement. This may be attributed to the laser’s photocoagulation effect, which, despite allowing excellent hemostasis, can provoke a unique initial inflammatory response [22].

Functional outcomes assessed by the Tongue Test mirrored these trends, with both groups showing marked improvement immediately after surgery (D0-post), stability at D7, and further gains by D14. These findings suggest that tissue healing occurring within the first two weeks post-surgery significantly contributes to enhanced tongue function, regardless of the surgical technique used [23, 24].

Pain assessment using the CRIES scale and monitoring of analgesic use revealed no significant differences between groups, indicating both methods provided similar perioperative comfort. Both groups showed a significant reduction in pain shortly after the procedure. Interestingly, literature in adult populations suggests lasers may offer superior outcomes in terms of postoperative pain and scarring [25, 26], but this was not observed in our study—possibly due to differences in age-related healing dynamics and the challenge of accurately assessing pain in non-verbal infants. Regarding anesthetic protocol, although no adverse effects were observed, clinicians should carefully weigh the risks and benefits of topical benzocaine use in infants, following national regulations and considering safer alternatives where appropriate.

Healing quality, evaluated with the Landry healing index, improved progressively in both groups. Despite known laser-associated benefits such as photobiomodulation, disinfection, and hemostasis, no statistically significant differences were noted in healing scores. However, a negative correlation was identified between the total energy applied during laser surgery and healing quality at day 14, suggesting that excessive thermal energy may impair tissue repair [15]. This aligns with existing literature reporting thermal damage as a concern with high laser energy levels [14, 22, 25, 26].

Weight gain, an indirect marker of feeding success, was also monitored. Both groups demonstrated progressive body mass increase, supporting the idea that surgical treatment of ankyloglossia can resolve sucking difficulties and enhance breast milk intake. No significant differences in weight gain were found between the SC and DL groups [2, 18, 27].

Breastfeeding performance was assessed using the LATCH and IBFAT protocols, which measure sucking efficiency and infant satisfaction [28, 29]. On day 7, the SC group showed significantly better results, possibly due to faster lingual functional recovery and less fibrous tissue formation compared to DL [14]. By day 14, however, the difference resolved, indicating similar long-term outcomes. Previous research supports the idea that when tongue function is restored, the choice of surgical method may not influence long-term breastfeeding success [30].

Although no significant differences were observed between the groups for most parameters, functional breastfeeding performance showed a temporary advantage for the scalpel group on the 7th postoperative day. However, this difference was not sustained by day 14, indicating that both surgical methods offer comparable clinical efficacy in the medium term. A noteworthy finding was the negative correlation between the total energy applied during the diode laser procedure and the healing index on the 14th day, suggesting that higher energy doses may negatively affect tissue repair. Despite this, both techniques resulted in improved tongue function, reduced pain, and progressive weight gain during the postoperative period.

From a clinical perspective, the success of either surgical technique depends not only on the choice of instrument but also on the clinician’s training and adherence to evidence-based procedural principles. Proper assessment should begin with a comprehensive intraoral examination to identify all restricted tissues and evaluate functional limitations, ensuring that surgical intervention is truly indicated. An accurate diagnosis, ideally combining anatomical and functional criteria, guides appropriate case selection. Regardless of whether a scalpel or diode laser is used, the procedure should achieve a complete release of the restrictive tissue, ideally creating a diamond-shaped, lay-flat wound to optimize mobility and reduce the risk of reattachment. Postoperative care is equally critical, including scheduled follow-up to monitor healing and function, as well as referral to a speech-language pathologist for guidance on appropriate exercise protocols. Adhering to these steps maximizes the likelihood of achieving optimal functional outcomes for patients and minimizing recurrence.

This study provides meaningful insight into the clinical outcomes of two surgical techniques for the treatment of ankyloglossia in infants. By comparing the scalpel and diode laser under standardized conditions, it offers valuable evidence to guide clinical decision-making. However, it has limitations that should be acknowledged: the sample size, although clinically relevant, may not be sufficient to detect subtle differences between groups or to generalize the findings to broader populations; the short follow-up period limits the evaluation of long-term outcomes related to tongue function, scarring, and breastfeeding success, and future studies with extended follow-up (3–6 months) would be valuable to assess the persistence of surgical results and identify potential late complications, such as scar formation or recurrence; the assessment of pain relied on indirect scales, which may be subject to subjectivity and variability, especially in infants who cannot self-report. Additional studies with larger sample sizes, extended follow-up, and objective photographic assessments are encouraged to validate and expand upon these findings.

## Conclusions

Both the conventional scalpel and diode laser proved to be safe and effective for improving tongue mobility, feeding function, and overall well-being in infants. A temporary advantage in breastfeeding function was observed with the scalpel in the early postoperative period, whereas further investigation is needed to optimize diode laser parameters and assess their impact on healing. Technique selection should be individualized, considering available resources, practitioner expertise, and parental preferences.

## Data Availability

The datasets used and/or analysed during the current study are available from the corresponding author on reasonable request.

## Abbreviations

SC: Scalpel
DL: Diode Laser
BTAT: Bristol Tongue Assessment Tool
Tongue Test: Functional and morphological assessment protocol of tongue mobility
CRIES: Crying, Requires oxygen, Increased vital signs, Expression, Sleepless
LATCH: Latch, Audible swallowing, Type of nipple, Comfort, Hold
IBFAT: Infant Breastfeeding Assessment Tool
DO-pre: Day of surgery (baseline preoperative)
DO-intra: Day of surgery (intraoperative)
DO-post: Day of surgery (immediate postoperative)
DO-30post: Day of surgery (30 minutes post-operative)
D7: 7th postoperative day
D14: 14th postoperative day
J: Joules
r: Pearson’s correlation coefficient
